# Cerebral ischemia induces TRPC6 via HIF1α/ZEB2 axis in the glomerular podocytes and contributes to proteinuria

**DOI:** 10.1038/s41598-019-52872-5

**Published:** 2019-11-29

**Authors:** Krishnamurthy Nakuluri, Rajkishor Nishad, Dhanunjay Mukhi, Sireesh Kumar, Venkata P. Nakka, Lakshmi P. Kolligundla, Parimala Narne, Sai Sampath K. Natuva, Prakash Babu Phanithi, Anil K. Pasupulati

**Affiliations:** 1 Department of Biochemistry, University of Hyderabad, Hyderabad, 500046 India; 2 Department of Biotechnology & Bioinformatics, University of Hyderabad, Hyderabad, 500046 India; 3 Department of Biochemistry, Acharya Nagarjuna University, Guntur, 522510 India; 4 Narayana Medical College, Nellore, 524003 India

**Keywords:** Stress fibres, Podocytes

## Abstract

Podocytes are specialized cells of the glomerulus and key component of the glomerular filtration apparatus (GFA). GFA regulates the permselectivity and ultrafiltration of blood. The mechanism by which the integrity of the GFA is compromised and manifest in proteinuria during ischemic stroke remains enigmatic. We investigated the mechanism of ischemic hypoxia-induced proteinuria in a middle cerebral artery occlusion (MCAO) model. Ischemic hypoxia resulted in the accumulation of HIF1α in the podocytes that resulted in the increased expression of ZEB2 (Zinc finger E-box-binding homeobox 2). ZEB2, in turn, induced TRPC6 (transient receptor potential cation channel, subfamily C, member 6), which has increased selectivity for calcium. Elevated expression of TRPC6 elicited increased calcium influx and aberrant activation of focal adhesion kinase (FAK) in podocytes. FAK activation resulted in the stress fibers reorganization and podocyte foot process effacement. Our study suggests overactive HIF1α/ZEB2 axis during ischemic-hypoxia raises intracellular calcium levels via TRPC6 and consequently altered podocyte structure and function thus contributes to proteinuria.

## Introduction

Extreme physiological and pathological conditions impose challenges on human physiology. The normal functioning of the human body demands both continuous and adequate supply of oxygen whereas relative (hypoxia) and the absolute deficiency (anoxia) of oxygen are a risk to human health. Human organs vary in their oxygen dependency and susceptibility to oxygen deficiency. Brain and kidney are most hypoxia-sensitive organs. Oxygen is involved in the formation of ATP from ADP and ATP-dependent active salt reabsorption in kidney demands high oxygen supply^1^. Kidney carries out its functions within a narrow range of partial pressure of oxygen, which is very low in the inner medulla (5 mmHg) compared with the outer cortex (50 mmHg)^2^. Furthermore, renal vasculature despite its low-resistance subjected to continuous perfusion^3,4^. Vascular architecture of the kidney and surplus demand for oxygen together let the kidneys highly sensitive to oxygen-deprived conditions^1,5,6^. Limitations in oxygen supply impose kidneys to undergo hypoxia-induced maladaptation, which likely reflects in the pathophysiology of acute kidney injury and proteinuria^6–12^.

The vertebrate kidneys regulate homeostasis predominantly by controlling acid-base, electrolyte, and water balance. Kidneys are also instrumental in ultrafiltration of plasma components and regulating the composition of urine. Proteinuric condition suggests abnormalities in the glomerular filtration apparatus (GFA)^13^. Three layers of GFA are podocytes, glomerular basement membrane (GBM), and perforated endothelium^13^. Clinical conditions such as stroke and sleep apnea are associated with proteinuria and are presented with reduced renal perfusion and moderate to severe hypoxia^12,14^. Accumulated evidence suggests that hypoxia contributes to the proteinuria and pathogenesis of chronic kidney disease (CKD)^6,7,10,15–17^. The prevalence of CKD is more than 30% among stroke subjects^18^. Renal dysfunction is a worse clinical outcome in patients with ischemic stroke^19,20^ and it is an independent predictor of stroke mortality^18^.

Epidemiological studies revealed that almost 1 in 10 adults in the USA has proteinuria^21^. Owing to the large volume of proteinuric population and the incidence of proteinuria with the disorders wherein anoxia/hypoxia prevail, it is crucial to gain insights on the pathophysiological relation between proteinuria and hypoxia. Middle cerebral artery occlusion (MCAO) model is routinely employed to mimic human ischemic stroke. Using the MCAO model we investigated the mechanism of glomerular dysfunction in ischemic stroke rats. We found that ischemic stroke and the resultant hypoxic injury manifested in the elevated expression of HIF1α in various organs including kidney. We observed elevated expression of ZEB2 and TRPC6 in glomerular podocytes from ischemic stroke rats. Elevated intracellular calcium levels were observed in podocytes with increased TRPC6 expression. Calcium-induced FAK activation resulted in stress fibers rearrangement and manifested in impaired podocyte structure and function. Overactivity of the HIF1α/ ZEB2 axis and TRPC6 could be a mechanism by which systemic hypoxia manifests in podocyte injury and proteinuria.

## Results

### The ischemic stroke resulted in systemic hypoxia and proteinuria

**Figure 1:**
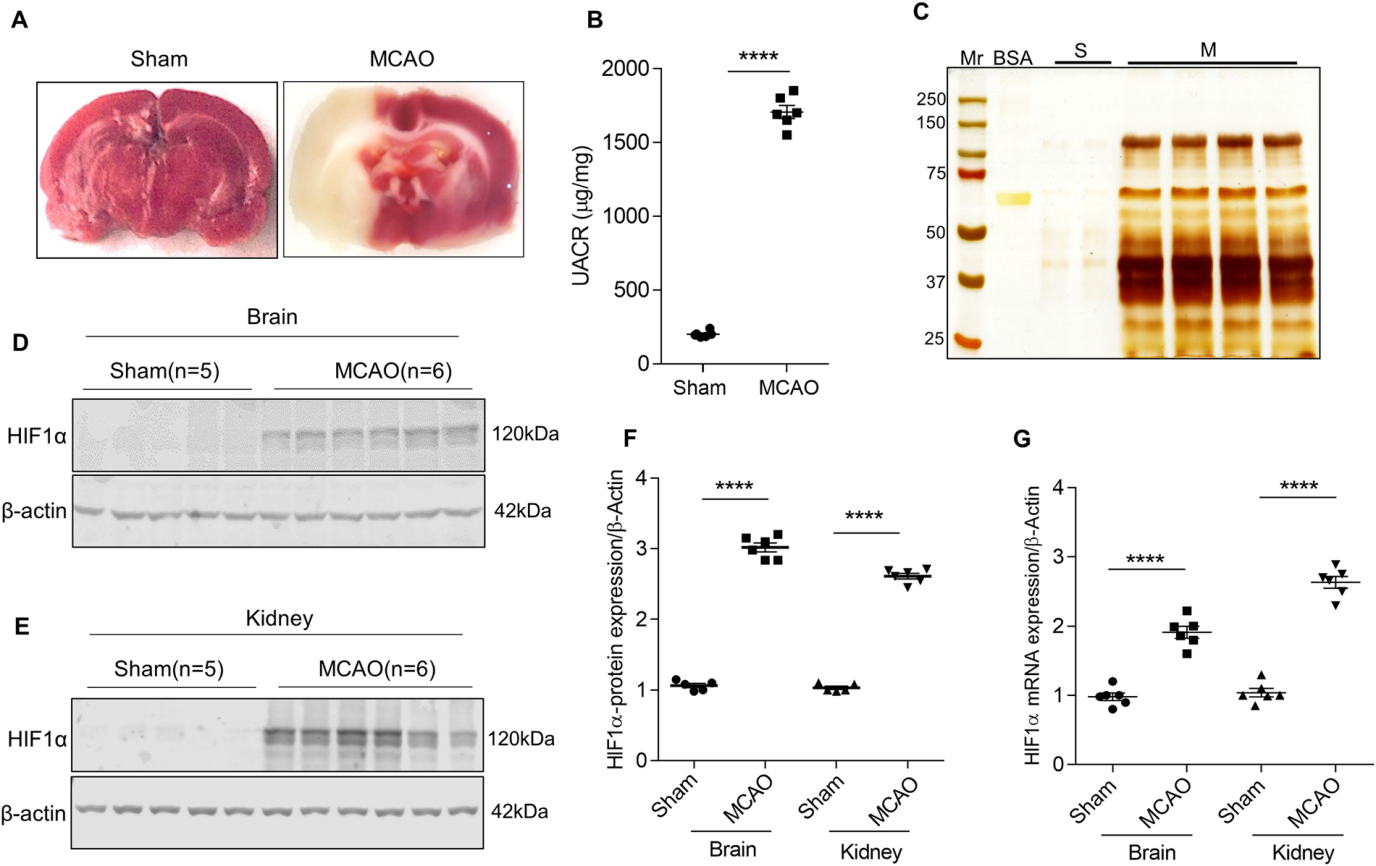
Ischemic stroke alters kidney function. (**A**) TTC staining images of sham and ischemic stroke-induced (MCAO) rat brain. (**B**) Estimation of albumin and creatinine levels in MCAO rats. Error bars indicate mean ± SE; n = 6. ****p < 0.0001. (**C**) Urine samples from sham (S) and stroke-induced rats (M) were subjected to SDS-PAGE and urinary proteins were visualized by silver staining, Mr, molecular weight marker (#1610374; Bio-Rad); BSA, Bovine serum albumin. HIF1α expression in the infarcted region of the brain (**D**) and glomerular lysates (**E**) from sham and MCAO rats. Densitometric analysis of HIF1α band is depicted after normalized for respective β-actin expression (**F**). Error bars indicate mean ± SE; n = 5–6. ****p < 0.0001. (**G**) Steady-state mRNA levels of HIF1α from the brain and glomerular lysates were measured by qRT-PCR. Error bars indicate mean ± SE; n = 6. ****p < 0.0001.

We performed triphenyl tetrazolium chloride (TTC) staining to assess the infarct size and injury following MCAO and reperfusion. Ischemic region of the brain showed white color when stained with TTC indicating the infarct lesions (Fig. 1A). 24 h following the MCAO surgery in rats, we measured the urine albumin to creatinine ratio (ACR) to evaluate the effect of ischemic stroke injury on renal function. The ischemic stroke resulted in significant albuminuria compared with sham-operated rats (Fig. 1B). Silver staining of SDS-PAGE gels revealed a significant amount of protein (particularly albumin) in urinary fractions from stroke-induced rats compared with sham-operated rats (Fig. 1C). We analyzed the expression of HIF1α in brain and kidney to ascertain whether ischemic-reperfusion injury elicits hypoxia in distant organs. Interestingly, we found that expression of HIF1α is elevated in the kidney in addition to the brain (Fig. 1D–G). The data suggest ischemic stroke resulted in renal hypoxia and is evidenced by elevated expression of HIF1α.

**Figure 2:**
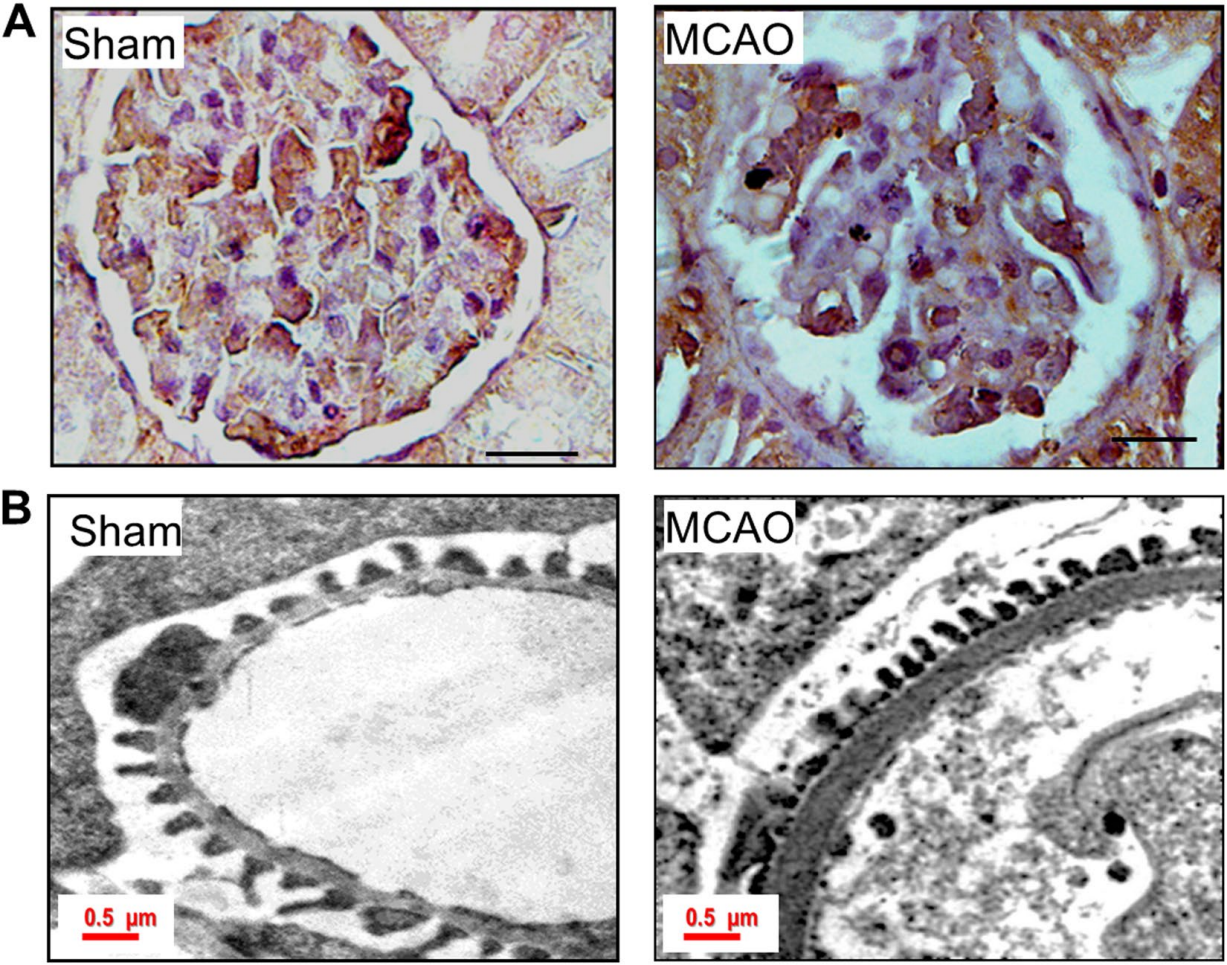
Ischemic-hypoxia elicits podocyte injury. (**A**) Staining for WT1 in sham and MCAO rat glomerular sections. The scale bar represents images of 10 µm and images were captured with a 100x objective of Leica trinocular microscope. (**B**) TEM images of podocyte foot processes in sham and MCAO rat kidney sections. In ischemic-stroke rats, podocyte foot-processes were small and the thickness of GBM was increased compared to sham-operated rats.

Since we observed proteinuria during ischemic stroke, we performed various staining procedures to assess renal morphology in these rats. PAS staining did not reveal glomerulosclerosis in stroke-induced rats. Owing to the importance of podocytes in glomerular filtration we assessed the podocyte number and their morphology. WT1 staining revealed a decreased number of podocytes in stroke-induced rats (Fig. 2A). Transmission electron microscope images revealed shortened podocyte foot-processes and an increase in the thickness of the basement membrane (Fig. 2B).

### Ischemic hypoxia induces ZEB2 and TRPC6 expression in podocytes

**Figure 3:**
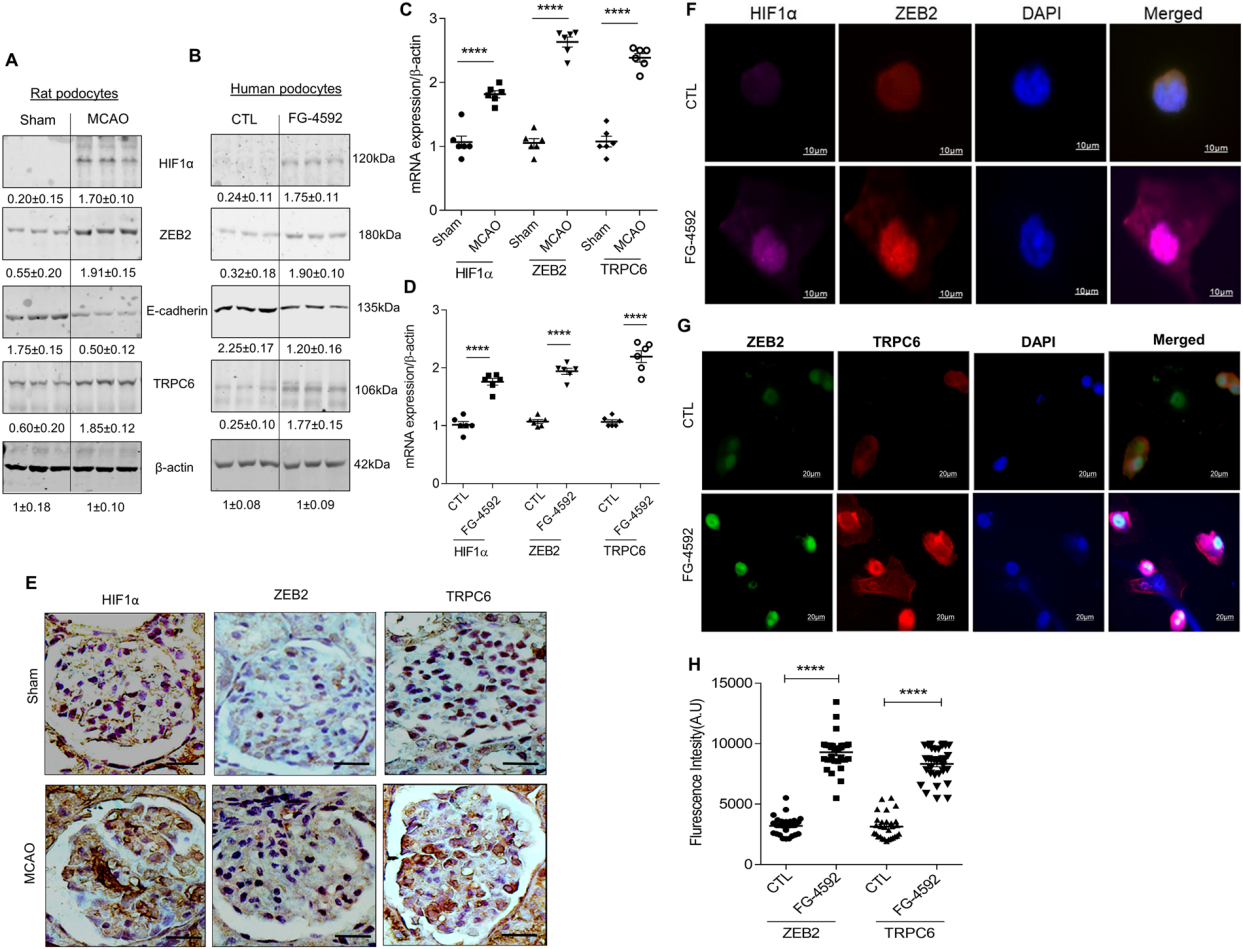
Ischemic-hypoxia induces ZEB2 and its target genes in glomerular podocytes: (**A**) Lysate from primary podocyte isolated from sham and MCAO rat kidney was used to assess the expression of HIF1α, ZEB2, E-cadherin, and TRPC6. Fold change values are mentioned under each blot. (**B**) Differentiated human podocytes treated with or without FG-4592 and analyzed the expression of HIF1α, ZEB2, E-cadherin and TRPC6. Fold change values are mentioned under each blot. The steady-state abundance of mRNA levels of HIF1α, ZEB2, and TRPC6 from podocytes isolated from sham and MCAO rats (**C**) and human podocytes treated with or without FG-4592 (**D**). Error bars indicate mean ± SE; n = 6. ****p < 0.0001. (**E**) Immunohistochemical analysis of HIF1α, ZEB2, and TRPC6 in glomerular sections from sham and MCAO rats. The scale bar represents images of 10 µm and images were captured with a 100x objective of Leica trinocular microscope. (**F**) Co-localization of HIF1α and ZEB2 in human podocytes treated with or without FG-4592. Images were acquired using a Zeiss 100x objective. (**G**) Elevated expression of ZEB2 and TRPC6 in human podocytes treated with FG-4592. Images were acquired with a Leica trinocular 63x objective and (**H**) fluorescence intensity of ZEB2 and TRPC6 expression in podocytes treated with or without FG-4592. Error bars indicate mean ± SE; n = 20. ****p < 0.0001.

ZEB2 expression is concomitant with HIF1α accumulation in podocytes exposed to hypoxia^22^. ZEB2, a zinc-finger transcription factor regulates the expression of several proteins representing various cellular processes including cell adhesion and epithelial-mesenchymal transition (EMT). Interestingly, in stroke-induced rats, we observed elevated expression of ZEB2 along with HIF1α in the glomerular podocytes (Fig. 3A,C,E). We observed reduced expression of E-cadherin in podocytes isolated from MCAO rats and human podocytes treated with FG-4592 (Fig. 3A–D). FG-4592 is a prolyl hydroxylase inhibitor, which stabilizes the HIF1α expression and elicits activation of target genes and signaling events. E-cadherin is a bona fide target of ZEB2 and attenuation of E-cadherin is a characteristic feature of EMT^23–25^. Loss of ZEB2 in epithelial cells showed migration defects and also presented with decreased expression of TRPC6 (transient receptor potential cation channel, subfamily C, member 6)^26^. Promoter analysis (TFSearch) showed that ZEB2 occupies E2-box 1 (-430′CAGGTG′-425) of the of human TRPC6 promoter. Further, we also found that TRPC6 expression elevated in both stroke-induced rat podocytes and human podocytes exposed to FG-4592 (Fig. 3A–D). Immunostaining data revealed that increased expression of HIF1α, ZEB2, and TRPC6 in glomeruli from stroke-induced rats (Fig. 3E). Immunofluorescence data suggests co-localization of HIF1α and ZEB2 in podocytes treated with FG-4592 (Fig. 3F). Further, we also demonstrated elevated expression of ZEB2 and TRPC6 in FG-4592 treated podocytes (Fig. 3G,H). Taken together, our data suggest TRPC6 expression is concomitant with ZEB2 expression in podocytes with elevated HIF1α expression.

### ZEB2 induces TRPC6 expression and calcium influx in podocytes

**Figure 4:**
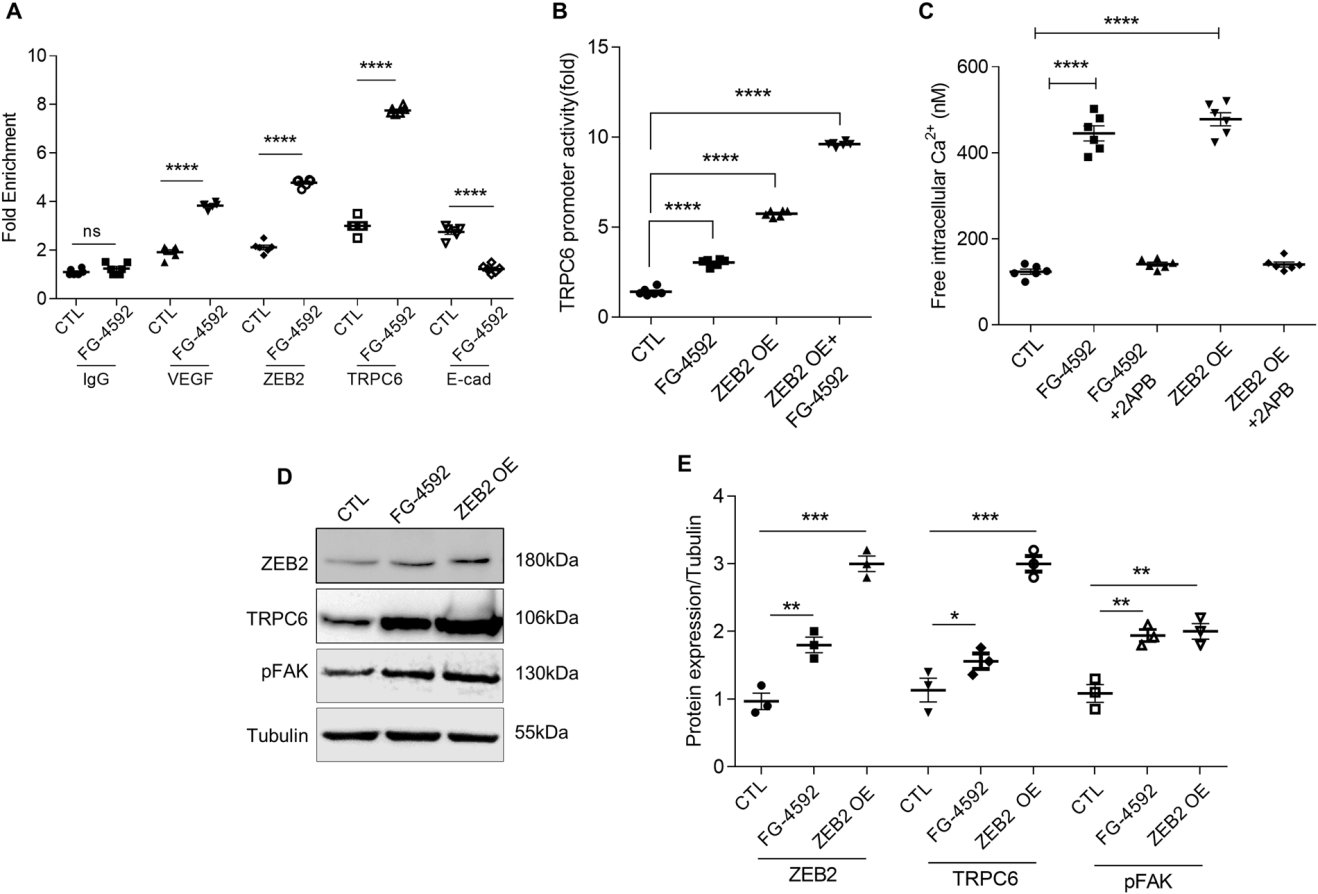
Hypoxia induces HIF1α-ZEB2-TRPC6 axis: (**A**) ChIP analysis with chromatin fractions from podocytes exposed to FG-4592 was performed as described in methods. Input DNA and DNA from each of the immunoprecipitated samples were PCR amplified for hypoxia response element (HRE) in VEGF promoter and ZEB2 promoter and E2-box region in both TRPC6 promoter and E-cadherin promoter. HRE in VEGF promoter fragment and E2-box in E-cadherin promoter serve as positive controls for HIF1α binding and ZEB2 binding respectively. Error bars indicate mean ± SE; n = 6. **p < 0.008 and ***p < 0.0004. (**B**) TRPC6 promoter activity was measured in HEK293T cells that ectopically expressing ZEB2 and exposed to FG-4592. Renilla luciferase was used as an internal control to normalize transfection efficiency. Error bars indicate mean ± SE; N = 6. ****p < 0.0001. (**C**) Intracellular free calcium levels in podocytes were measured by Fluo3-AM following treatment with FG-4592 in the presence or absence of 2-APB. Calcium levels were measured in podocytes that ectopically express ZEB2 and treated with or without 2-APB. Error bars indicate mean ± SE; n = 6. ****p < 0.0001. (**D**) Immunoblot analysis of ZEB2, TRPC6, and pFAK in podocytes treated with FG-4592 or ectopically expressing ZEB2 (ZEB2 OE). (**E**) Quantification of band intensities of ZEB2, TRPC6, pFAK was ImageJ analysis (NIH). Error bars indicate mean ± SE; n = 3. *p < 0.01,**p < 0.008, and ***p < 0.0002.

We performed ChIP assay to demonstrate the interaction between TRPC6 promoter and ZEB2. We found that ZEB2 binds to the proximal promoter of TRPC6 at the E2-box 1 region during hypoxic conditions (Fig. 4A). E-cadherin promoter has putative ZEB2 binding site, therefore we used E-cadherin as a positive control^23^. We also confirmed HIF1α occupancy on ZEB2 promoter (Fig. 4A) wherein, the interaction between HIF1α with VEGF promoter serves as a positive control. Furthermore, we observed increased TRPC6 promoter activity in HEK293T cells treated with either FG-4592 (Fig. 4B) or with ectopic expression of ZEB2.

Upon confirming that HIF1α/ZEB2 axis elicits TRPC6 expression, we investigated the significance of elevated expression of TRPC6 in podocytes. Although TRP family proteins have an affinity for cation transport, TRPC6 has selectivity for calcium influx. Among glomerular cells, TRPC6 expresses predominantly in podocytes^27^. We found increased intracellular calcium influx in podocytes exposed to FG-4592 as measured by calcium-sensitive fluorescent dye Fluo-3AM (Fig. 4C). While ectopic expression of ZEB2 increased calcium influx, 2-aminoethoxy diphenylborate (2APB, a calcium channel blocker) attenuated intracellular calcium levels in podocytes (Fig. 4C).

### ZEB2 regulates activation of FAK via TRPC6

**Figure 5:**
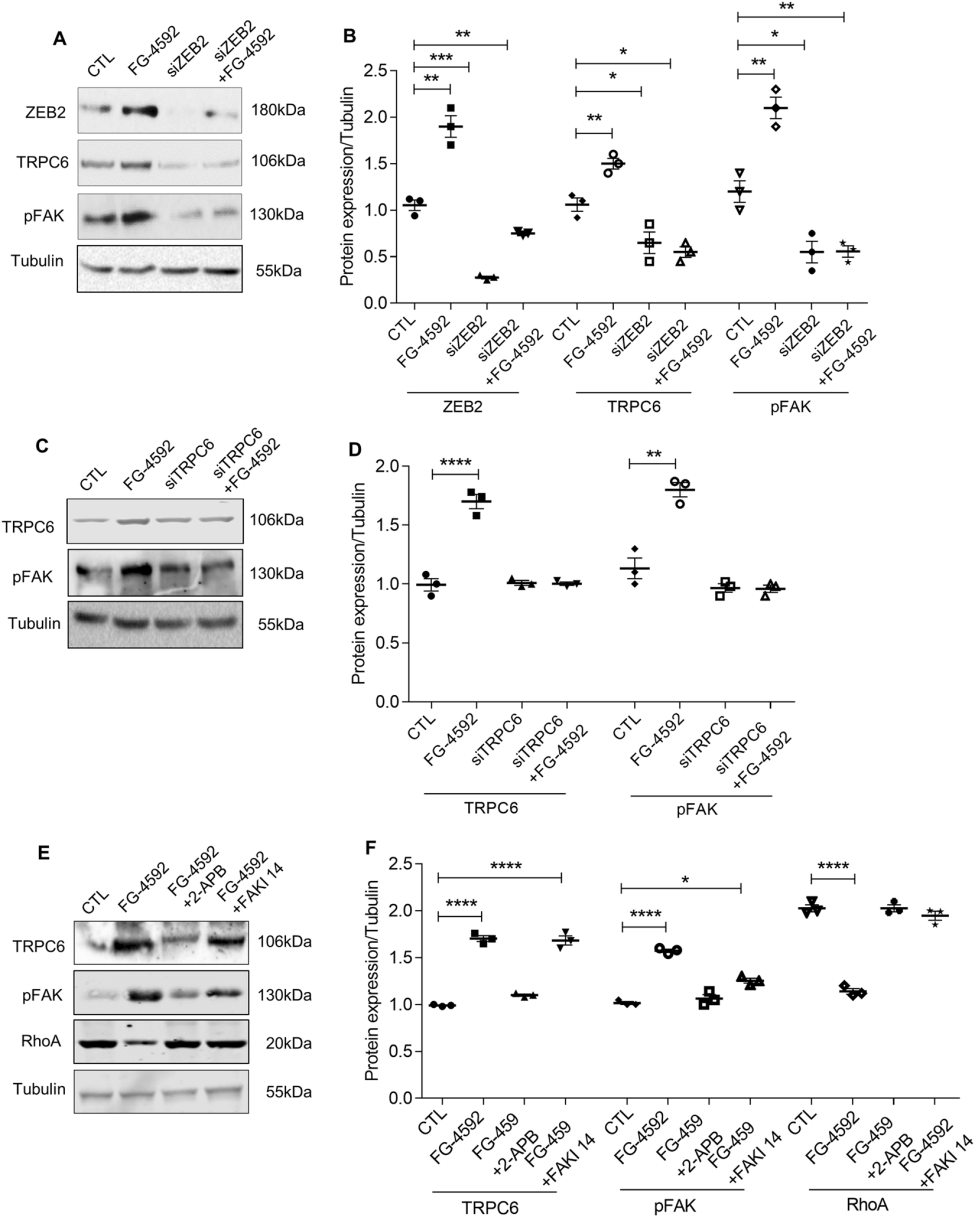
Essential role of ZEB2 in regulating TRPC6 expression: (**A**) Immunoblotting analysis of ZEB2, TRPC6, and pFAK expression in podocytes expressing siZEB2 and treated with or without FG-4592. (**B**) Quantification of band intensities of ZEB2, TRPC6, pFAK was performed with ImageJ software. Error bars indicate mean ± SE; n = 3, *p < 0.01, **p < 0.008, and ***p < 0.0002. (**C**) Immunoblotting analysis of TRPC6 and pFAK expression in podocytes in which TRPC6 expression was knocked-down and treated with or without FG-4592. (**D**) Quantification of band intensities of western blots was performed with ImageJ. Error bars indicate mean ± SE; n = 3, **p < 0.008 and ****p < 0.0001. (**E**) Immunoblotting analysis of TRPC6, pFAK, and RhoA expression in podocytes exposed to FG-4592 and treated with or without 2APB and FAKI14. (**F**) Quantification of band intensities of western blots was performed with ImageJ. Error bars indicate mean ± SE; n = 3, *p < 0.01 and ****p < 0.0001.

Elevated intracellular calcium levels elicit auto-phosphorylation (Y397) of focal adhesion kinase (FAK)^28^. On the other hand, inhibition of FAK protects against effacement of podocyte foot-processes^29^. FAK is a central protein of focal adhesions and it regulates the function of several cytoskeletal and focal adhesion proteins^30^. Therefore, we measured pFAK levels in podocytes ectopically expressing ZEB2 or treated with FG-4592. ZEB2 overexpression in podocytes resulted in both increased TRPC6 expression and activation of FAK (Fig. 4D,E). On the other hand, ZEB2 knockdown resulted in reduced expression of TRPC6 and pFAK (Fig. 5A,B). Further, to ascertain the essential role of TRPC6 in FAK activation, we attenuated TRPC6 expression by siTRPC6 and measured the pFAK levels. We observed pFAK levels are proportional with TRPC6 levels in cells treated with or without FG-4592 (Figs. 5C,D and S1, 2). Together, the data suggest that ZEB2 regulates phosphorylation of FAK via TRPC6.

Activated FAK is localized to focal adhesions. Focal adhesions serve as connections between the cytoskeleton and the cell matrix. Formation of focal adhesions is regulated by RhoA^30^. FAK suppresses RhoA activity and promotes focal adhesion turnover^30,31^. We noticed decreased RhoA while FAK gets activated in podocytes treated with FG-4592 (Fig. 5E). It is noteworthy that both calcium channel blocker (2APB) and FAK inhibitor14 (FAKI14) (Fig. 5E,F) ameliorated RhoA expression in podocytes treated with FG-4592 (Fig. 5E,F).

### HIF1α alters podocyte actin cytoskeleton

**Figure 6:**
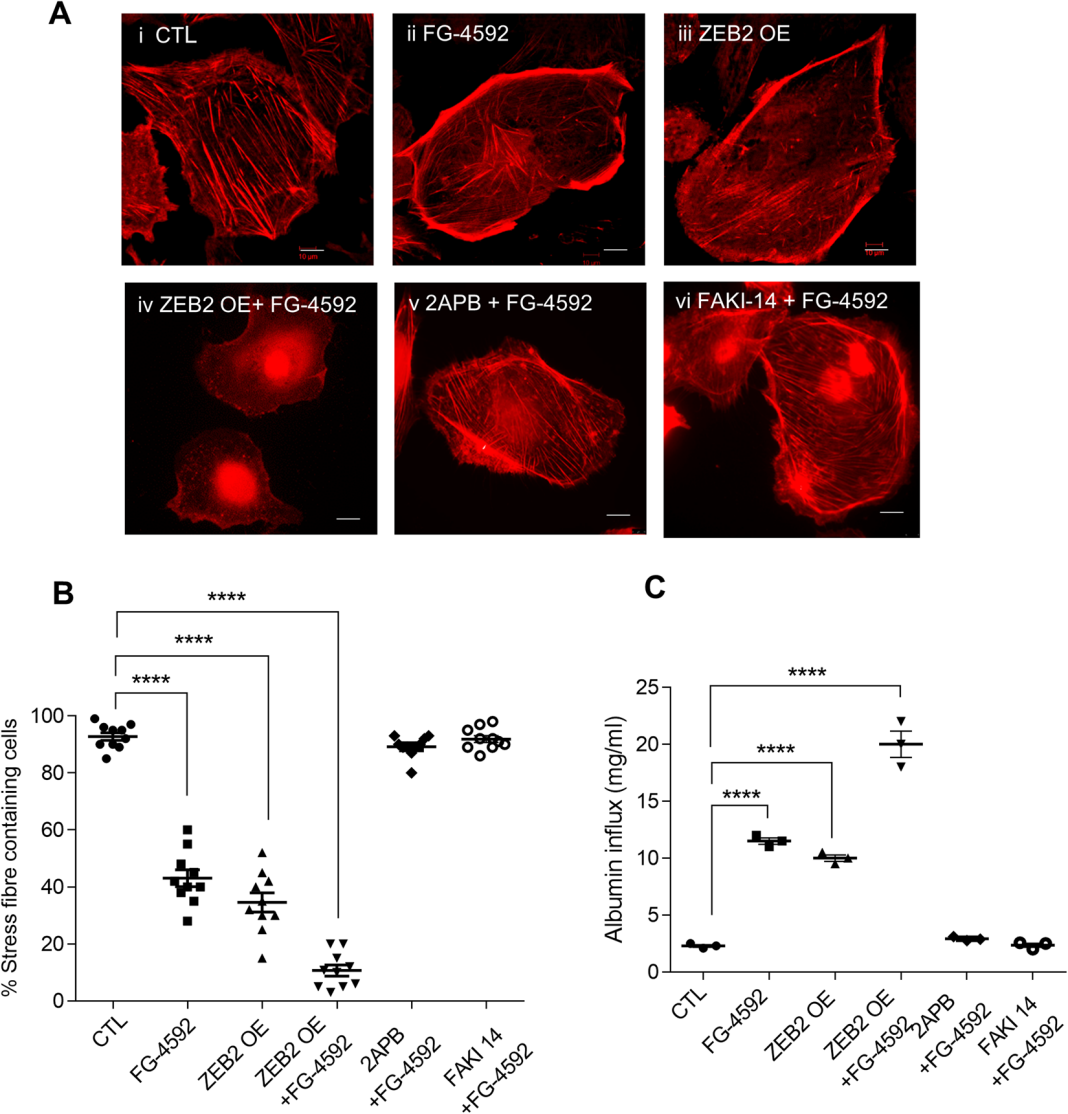
HIF1α-ZEB2-TRPC6 axis regulates podocyte cytoskeleton reorganization: (**A**) Phalloidin staining was performed to visualize intracellular F-actin stress fibers (SFs) in podocytes. Podocytes treated with FG-4592 (ii) compromised their morphology and SFs arrangement compared with cells naïve to FG-4592 (i). Ectopic expression of ZEB2 with and without FG-4592 treatment also resulted in an altered SFs arrangement (iii & iv). Calcium channel blocker; 2APB or FAK inhibitor; FAKI14 (v & vi) ameliorated FG-4592 induced SFs reorganization. The scale bar represents images of 50 µm and images were captured with a 63x objective of Leica trinocular microscope. (**B**) The percentage of stress fibers containing podocyte cells were quantified and analyzed by Image-J (NIH). Error bars indicate mean ± SE; n = 10 podocyte cells in each condition. ****p < 0.0001. (**C**) Quantification of albumin influx across podocyte monolayer. Podocytes transduced with or without ZEB2 were grown as a monolayer on cell culture insert and allowed to differentiate. Differentiated podocytes were exposed to FG-4592 for 24 h and treated with FAKI14 or 2APB and albumin influx assay was performed as described in methods. Error bars indicate mean ± SE; n = 3. ****p < 0.0001.

As we noticed activation of FAK and decreased RhoA expression in podocytes with elevated HIF1α, we next investigated for the cytoskeletal abnormalities, if any. Stress fibers are bundles of actin filaments held together by actin-crosslinking proteins such as α-actinin, fascin, espin, and filamin^32^. We assessed the distribution of actin stress fibers in cells that are naïve or exposed FG-4592 employing phalloidin staining. Differentiated podocytes are naïve to hypoxia exhibit orderly arranged non-branching stress fibers (Fig. 6A–i). Upon exposure to hypoxia, the orderly arranged stress fibers of the podocyte actin cytoskeleton were disrupted (Fig. 6A–ii). Ectopic expression of ZEB2 resulted in disrupted stress fibers (Fig. 6A–iii). Altered morphology and severe loss of stress fibers were observed in podocytes that ectopically express ZEB2 and treated with FG-4592 (Fig. 6A–iv). Interestingly, 2APB and FAKI14 ameliorated stress fiber distribution in FG-4592 treated podocytes (Fig. 6A–v&vi). We quantified the number of stress fibers per podocyte and the ratio of stress fibers to total cell size was depicted (Fig. 6B). The data suggest that ZEB2 overexpression alters podocyte cytoskeleton whereas calcium channel blocker or FAK inhibitor ameliorate HIF1α induced podocyte cytoskeletal rearrangements. The disruption of stress fibers of the podocyte actin cytoskeleton may suggest the reason for altered cell morphology and FPE in podocytes from stroke-induced rats (Figs. 2B vs. 6A,B).

### Calcium channel blocker and FAK inhibitor prevent HIF1α-induced podocyte permeability

Earlier studies revealed that accumulation of HIF1α in podocytes increased podocyte permeability to albumin and blunting the expression of ZEB2 diminished the susceptibility podocyte monolayer to hypoxia-driven elevated albumin influx^22^. This data strongly suggest that ZEB2 is necessary to elicit HIF1α’s action on podocyte permeability. Since we showed that ZEB2 induces TRPC6 and calcium accumulation and consequent activation of FAK in podocytes, we assessed the permeability of podocytes treated with FG-4592 in the absence or presence of 2APB and FAKI14. Both 2APB and FAKI14 prevented the podocyte permeability to albumin (Fig. 6C), suggesting that prevention of calcium influx and/or inhibition of FAK preserve podocyte permselectivity during hypoxia.

### Co-expression of HIF1α, ZEB2, and TRPC6 in kidney diseases

**Figure 7:**
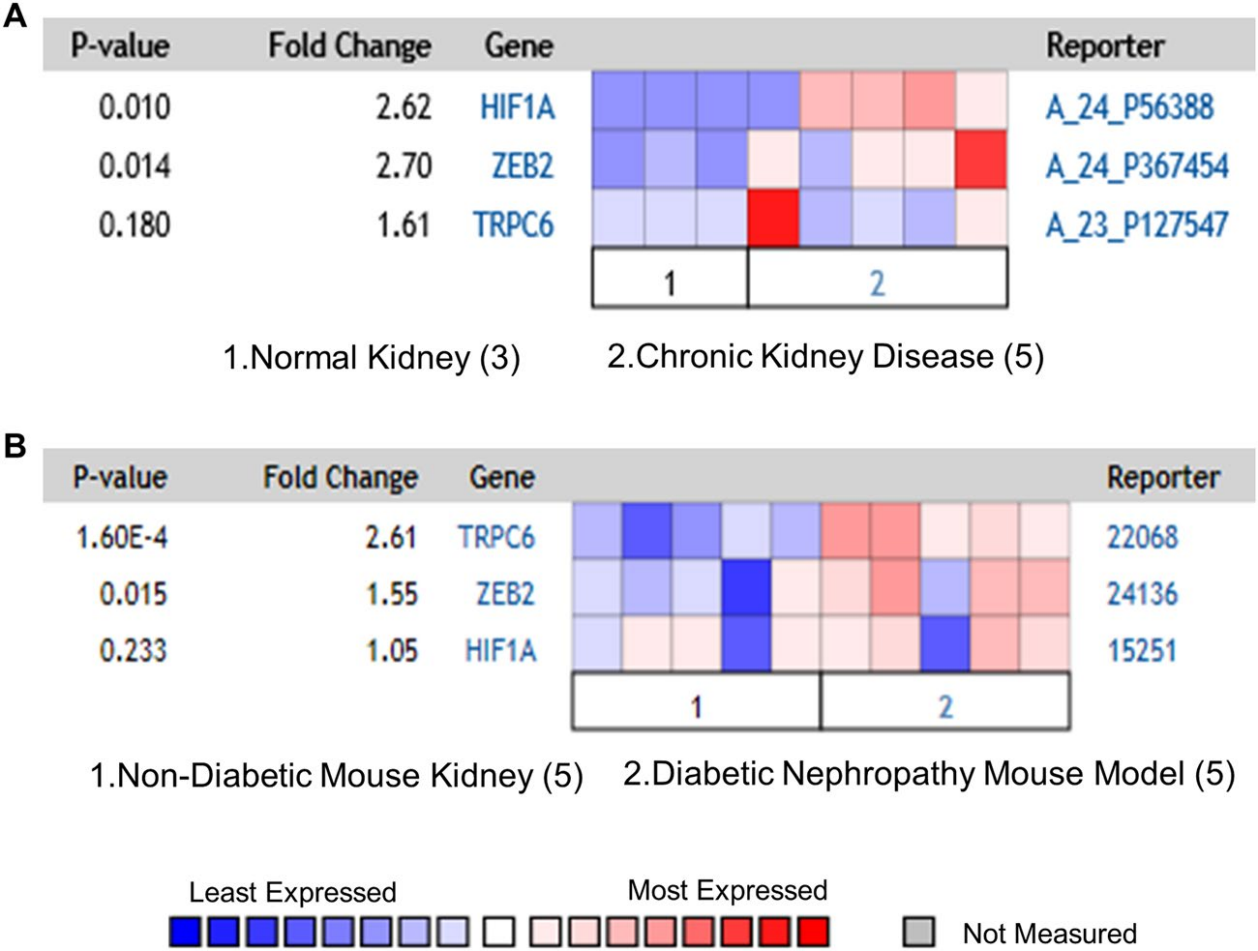
Co-expression of HIF1α, ZEB2, and TRPC6 in glomerular diseases. (**A**) Nakagawa CKD data set showing the elevated expression of HIF1α (2.6 fold), ZEB2 (2.7 fold), and TRPC6 (1.6 fold) in patients with chronic kidney disease vs. healthy kidney. (**B**) Hodgin diabetes mouse glomeruli datasets showing the elevated expression of ZEB2 (1.55 fold), and TRPC6 (2.61 fold) in mouse with diabetic nephropathy vs. non-diabetic mouse models. The data is obtained from *Nephroseq* (University of Michigan, Ann Arbor, MI).

It is noteworthy that intrarenal hypoxic injury is considered to be the common cause of renal dysfunction and proteinuria in conditions such as hypertension, diabetes, and stroke^33,34^. We performed co-expression analysis for HIF1α, ZEB2, and TRPC6 in the *Nephroseq* database (University of Michigan, Ann Arbor). *Nephroseq* analysis revealed increased expression of HIF1α, ZEB2, and TRPC6 in Nakagawa CKD dataset and Hodgin Diabetes Mouse Glomeruli datasets (Fig. 7A,B). The data suggests these three genes co-express in CKD of human origin and diabetic mouse glomerular diseases.

## Discussion

**Figure 8:**
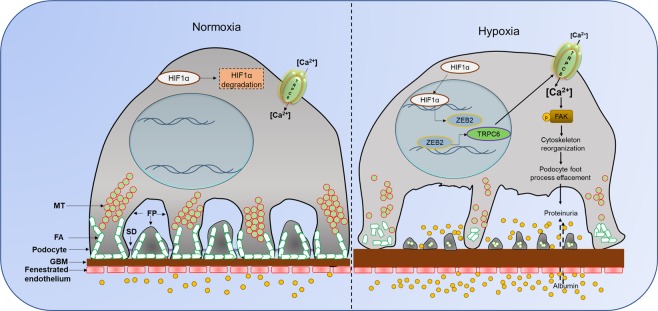
Proposed model for ischemic-hypoxia mediated podocyte injury. Ischemia-stroke rats develop systemic hypoxia that induces HIF1α accumulation in several susceptible sites including glomerular podocytes. HIF1α drives ZEB2 expression, which in turn induces TRPC6 expression. Elevated TRPC6 increases intracellular calcium levels and calcium-dependent phosphorylation of FAK elicits cytoskeletal rearrangements. These cytoskeletal rearrangements eventually manifest in the effacement of podocyte foot-processes and increased permeability to proteins and large molecules. The overactivity of the HIF1α/ZEB2/TRPC6 axis in podocytes elicits cytoskeletal abnormalities and proteinuria.

Podocytes are instrumental for contributing glomerular permselectivity and ultrafiltration of urine. It has been known that ischemic stroke is often associated with proteinuria. Owing to the importance of podocytes in glomerular filtration, we investigated the cellular effects of stroke-associated ischemia-hypoxia on podocyte biology. We show that following ischemic reperfusion, HIF1α and its down-stream target ZEB2 are elevated in the glomerular region and especially in podocytes. Our results suggest a novel role of HIF1α with the elevated expression of TRPC6 in podocytes. Elevated expression of TRPC6 is at least partially due to ZEB2 expression. TRPC6 ensures calcium influx into podocytes, which elicits FAK activation and these events culminate in the disruption of actin stress fibers. In addition to altered morphology of podocytes, accumulation of HIF1α resulted in the increased permeability to albumin across podocyte monolayer. Overall our results establish that TRPC6 is a novel target of HIF1α/ZEB2 axis and that transduces stroke-induced ischemia-hypoxia injury in podocytes (Fig. 8).

Rats underwent MCAO developed hypoxia as evidenced by the reduced partial pressure of oxygen (PaO2 ≤ 60%) and decreased oxygen saturation (SaO2 ≤ 80%) of arterial blood from 6 to 24 hours after reperfusion suggesting that these animals develop systemic hypoxia^35^. Average SaO2 levels were significantly lower in MCAO rats between 6 and 24hrs after reperfusion. MCAO is the most frequently used experimental model to mimic ischemic stroke and insufficient cerebral blood flow during ischemic stroke elicits hypoxic injury, which results in reduced arterial oxygen saturation^36^. We were interested in understanding the distant organ effect of stroke, particularly on glomerular function. Normally, synergy among arteriovenous oxygen shunting, renal blood flow, and glomerular filtration rate helps kidneys maintain arterial oxygen pressure at relatively stable levels^2^. This intricate interplay among several physiological factors makes kidneys susceptible to hypoxic injury^2^. It was reported that proteinuria is one of the major clinical outcomes following acute ischemic stroke^37^. Proteinuria refers to the impaired function of GFA; therefore, we investigated the effect of hypoxia in the glomeruli and in podocytes that are crucial to ensure glomerular permselectivity. Systemic mild hypotension is a feature of the MCAO model and mild hypotension does not affect systemic oxygen delivery to tissues^38,39^. Therefore, it appears that hypoxic effects in the MCAO group appear independent of hypotension. Although hypertension is an independent risk factor for the stroke and it also adversely affects glomerular filtration, the management of blood pressure during the stroke period has been controversial and remains uncertain^40^. While we can’t completely rule out the role of hypertension in GFA function, in this study, we specifically focused on the effect of hypoxic signaling on glomerular podocytes.

HIF1α is a major transcription factor that transduces an array of cellular processes to let the cells adapt to hypoxic injury. Our results establish that ischemic stroke-mediated stabilization of HIF1α induces the expression of ZEB2. It was also shown earlier that ZEB2 expression is elevated in podocytes exposed to normobaric hypoxia^41^. ZEB2 related to the δEF1 protein family and these proteins possess a homeodomain flanked by N- and C-terminal zinc finger clusters^42^. Canonically, ZEB2 is considered as a transcriptional repressor. E-cadherin is a well-known target for transcriptional suppression by ZEB2. Reduced E-cadherin expression is implicated with the transition of podocytes from epithelial to mesenchymal phenotype compromising their ability to provide epithelial coverage to glomerular capillaries^22,23^. However, in this study, we found that ZEB2 induces TRPC6 expression in podocytes. Similarly, in a recent study, it was shown that ZEB2 could also serve as a transcriptional activator^26,43^. It was reported that the upstream promoter of TRPC6 has several ZEB2 binding sites^26^. Together the data suggest that ZEB2 could be a dual transcription factor with both repressor and activator functions.

Based on their involvement in the pathology of several diseases TRP channels are considered as drug targets^44^. TRP channels are in general permeable to cations whereas, TRPC6 has more affinity for calcium and excess calcium influx is implicated several pathologies. Proline to glutamine substitution at position 112 (P112Q) enhances TRPC6-mediated calcium signals in response to agonist such as angiotensin II^45^. TRPC6 (P112Q) mutant is associated with focal segmental glomerulosclerosis. TRPC6 dependent calcium entry is implicated in late-onset of Alzheimer’s disease^46^. In podocytes, TRPC6 express in the cell body and foot processes and it interacts with slit-diaphragm proteins^47,48^. TRPC6 mediated calcium entry elicits albumin overload-induced ER stress and apoptosis in podocytes^49^. TRPC6 mediated calcium entry elicits albumin overload-induced ER stress and apoptosis in podocytes^49^. Other than TRP channels, cells express voltage-gated calcium channels (VGCC) and ligand-gated calcium channels (LGCC). VGCC are classically known as dihydropyridine channels, because of the presence of a dihydropyridine-binding site. It was shown that activation of endogenous TRPC6 channels increases cytosolic calcium levels and this response is blocked by nimodipine, a dihydropyridine class calcium channel blocker suggesting that the calcium entry via TRPC6, inurn activates VGCC^50^. It can be speculated that increased TRPC6 expression in podocytes may further increase calcium influx by downstream activation of VGCC. Increased function of TRPC6 channels in various cell types could directly or indirectly contribute to renal injury leading to fibrosis. Calcium entry through TRPC6 plays a necessary role for myofibroblast generation in response to stimulation by injury factors such as Ang-II and TGF-β^51^. Inhibition of TRPC6 channels offers renal protection specifically by ameliorating fibrosis^52^.

Podocyte actin cytoskeletal rearrangement is the common final pathway subsequently leads to podocyte foot process effacement (FPE)^53^. The contractile actin filament bundles that are highly ordered and arranged parallel in the foot processes were converted into disordered, short, and branched under pathological conditions, thus ensuring podocytes to compromise their unique structure. The disruption of stress fibers of the podocyte actin cytoskeleton could probably explain the reason for altered cell morphology and FPE in podocytes from ischemic reperfusion injury. Reversible FPE was observed in some transient proteinuric models that were generated by protamine sulfate infusion and lipopolysaccharide injection^54,55^. FAK is a non-receptor tyrosine kinase, which is recruited to focal adhesions by paxillin and talin^56^. FAK plays an essential role in cell motility, maintenance of cell morphology, and also regulates podocyte cytoskeleton. Podocyte-specific deletion of FAK in mice protected from podocyte injury and proteinuria^29^. A recent study suggests that podocyte injury activates FAK phosphorylation that elicits increased FAK turnover, FPE, and proteinuria^29^. FAK phosphorylation prompts podocyte injury, therefore inhibition of FAK signaling cascade may have therapeutic potential in the treatment of glomerular injury^29^. Alternatively, administration of calcium channel blockers improved renal function, GFR, renal blood flow, and electrolyte excretion^57,58^, since acute ischemic stroke induces intracellular calcium accumulation^59^. In summary, our study identified TRPC6 as a bona fide target of ZEB2 and transduces ischemia mediated podocyte injury and proteinuria. TRPC6 mediated calcium influx possibly mediate the podocyte cytoskeletal abnormality and calcium blockers could be a therapeutic option to combat ischemia-hypoxia injury in podocytes.

## Materials and Methods

### Intraluminal suture middle cerebral artery occlusion

Middle cerebral artery occlusion (MCAO) and sham surgery were performed in 8-week-old Male SD rats as reported earlier using the intraluminal monofilament technique^60^. Cerebral reperfusion was allowed by withdrawing the monofilament carefully after 2 h of surgery and animals were maintained for 24 h. The rats subjected to stroke and sham-surgery were transcardially perfused with saline under anesthetic conditions. Tissues were harvested and frozen for RNA and protein isolation whereas for fresh tissues were fixed for histological studies and TEM imaging. To assess the success of the model and infarct volume, 2-mm-thick coronal sections of the brain were prepared and stained with 1% triphenyltetrazolium chloride. University of Hyderabad’s Institutional animal ethics committee approved animal experiment protocols. We confirm that all methods were performed in accordance with the relevant guidelines and regulations.

### Urine analysis

Urine samples from rats were subjected to 10% SDS-PAGE gel and processed for silver staining as described earlier^22^. Urinary albumin (#11573) and creatinine (#11502) were measured using commercially available kits (Biosystems, Barcelona, Spain).

### Isolation of glomeruli and podocytes

The glomeruli from the kidneys were isolated by a series of stainless sieves as described earlier^22^. Primary podocytes from rat kidney were isolated as reported earlier^61^.

### Immunohistochemistry

Paraffin sections (5 µm) of kidney cortex were prepared with Leica microtome on to pre-coated glass slides. Sections were allowed for deparaffinization, rehydration, followed by antigen retrieval. Following permeabilization and blocking, sections were incubated with respective primary antibody at 4 °C for overnight. Further, incubation with secondary antibody and DAB staining with the kit method using Mouse/Rabbit PolyDetector DAB-HRP Detection kit (Santa Barbara, CA, USA). Images were captured with Trinocular microscope 100X objective (Leica, Buffalo Grove, IL).

### Transmission electron microscopy

Kidney sections were processed for TEM imaging as described in our earlier study^22^. Briefly, the renal cortex portion of the kidney from sham and stroke-induced rats were collected and fixed with 2.5% glutaraldehyde and 1% osmium tetroxide. Ultrathin sections were mounted on copper grids, stained with 3% uranyl acetate and images were obtained with JEM-1400TEM (Jeol, Peabody, MA).

### Cell culture

In this study, we employed human podocytes (A gift from Prof. Moin Saleem, University of Bristol) and HEK293T cells. Podocytes and HEK293T cells were cultured essentially as described earlier^22,23^.

### Immunoblotting

Equal concentration of protein from either glomerular or cell lysate was subjected to SDS-PAGE and blotted onto the nitrocellulose membrane. Immunoblotting and developing the blots was performed as reported earlier^22^.

### qRT-PCR analysis

Isolation of RNA, preparation of cDNA, and qRT-PCR were performed as reported earlier^41^. The expression level of each mRNA was normalized to β-Actin and quantified using the comparative Ct method. List of primers used in this study is provided in Table S1.

### Immunofluorescence

Podocytes were cultured on coverslips and allowed to differentiate. Following experimental conditions, these cells fixed with 4% paraformaldehyde and performed immunofluorescence protocol as reported earlier^22^. Imaging was done in a Leica trinocular fluorescent microscope under 60x oil objective.

### Transfection

HEK293T cells or differentiated podocytes were transfected using jetPEI reagent (Polyplus, Illkirch, France). 1 × 10^5^ cells were seeded per well in 6-well cell culture plates and transiently transfected with plasmid DNA or siRNA by mixing with NaCl-jetPEI complexes. After 48 hr of transfection, cells were washed twice with PBS and lysed with RIPA buffer; the expression levels were measured by western blotting as described above. Podocytes that ectopically express ZEB2 or ZEB2 knockdown were employed in calcium influx assay.

### Measurement of intracellular calcium

The protocol for measuring free intracellular calcium was adapted from modified AfCS procedure protocol^62,63^. Briefly, differentiated human podocytes were treated with or without calcium channel blocker (2-APB) for 2 h, followed by FG-4592 for 4 h. Cells were loaded with calcium sensitive Fluo3-AM and Pluronic F-127 (1:1) at a final concentration of 4 uM and 10%, respectively and incubated for 30 min in dark at 37 °C with 5%CO_2_. Cells were scraped using calcium free HBS solution and cell lysate was centrifuged at 400 × g for 5 min and supernatant was collected to measure fluorescence (F) (λex 485 nm; λem 538 nm). To obtain the total fluorescence of dye at saturating Ca^2+^, 1% NP-40 was added to release dye from cells and measured maximum fluorescence (F_ma*x*_). EGTA (0.5 M) was added to cell lysate and fluorescence of dye (F_min_) in the absence of free Ca^2+^ was obtained. We measured baseline fluorescence (F_basal_) of the dye in cell lysate naïve to FG-4592 and 2-APB. Free cytosolic calcium was quantified by the formula; [Ca^2+^] = F_basal_ − (K_d_ × ((F − F_min_)/(F_max_ − F))) where K_d_ for Fluo3-AM is 390 nM. Intracellular calcium was also measured in podocytes that overexpress ZEB2 in the presence or absence of 2APB.

### Phalloidin staining

Fluorescent phalloidin-TRITC conjugate staining was performed to visualize the distribution of stress fibers in differentiated podocytes essentially as described earlier^41^.

### ChIP assay

Approximately 80% of confluent podocytes were exposed to hypoxia. Chromatin immunoprecipitation (ChIP) was performed as described earlier^22^. Cells were cross-linked with formaldehyde and quenched with 125 mM glycine, then lysed with lysis buffer (1% SDS, 10 mMEDTA, 50 mM Tris-HCl, pH-8.1, protease inhibitors). Pre-clearing was performed with protein A/G beads followed by immunoprecipitation with the antibody of interest. Chromatin fragments were eluted from the beads and further purified by the phenol-chloroform method. This purified DNA was used for RT-PCR to quantify fold enrichment. The list of primers used in for ChiP experiments is provided in Table S2.

### Promoter-Reporter assay

TRPC6 promoter was cloned into pGL3 basic reporter vector with MluI and BglII restriction sites. The resultant pGL3-TRPC6 promoter construct was co-transfected with renilla plasmid into the HEK293T cells. Cells were then exposed to hypoxia or transfected with ZEB2 are scrapped with ice-cold PBS. Cells were collected after a spin down at 5,000 rpm for 5 min at 4 °C, followed by lysing the cells with a passive lysis buffer. 20 ul of lysate was used for measuring luciferase expression with LARII and renilla expression with stop glow reagents to quantify relative promoter activity.

### Albumin influx assay

Podocyte permeability was assessed by albumin influx assay as described earlier^22^. Briefly, human podocytes were allowed to differentiate as described above on collagen-coated transwell filters. Differentiated podocytes were exposed to hypoxia for 24 hrs and washed with phosphate buffer containing with 1 mM MgCl_2_ and 1 mM CaCl_2_. In the bottom chamber, 2 ml of RMPI 1640 medium containing 40 mg/ml BSA was added. In the top chamber, only RPMI 1640 (without albumin) was added. The concentration of BSA in the upper chamber was estimated at various time points.

### Statistical analysis

Statistical analysis was performed using GraphPad Prism V.7. Data are presented as mean ± S.E from two to three independent experiments (*in vitro* data) performed. Comparison between groups was performed using Student’s t-test p values equal to or less than 0.05 were considered significant.

## Supplementary information

Supplementary file
