# Supplementary material for: Cerebral ischemia induces TRPC6 via HIF1α/ZEB2 axis in the glomerular podocytes and contributes to proteinuria
Source: Sci Rep. 2019 Nov 29;9:17897. doi: 10.1038/s41598-019-52872-5 (PMC6884642; doi:10.1038/s41598-019-52872-5)
Supplement: Supplementary file 1 — Supplementary file [file 41598_2019_52872_MOESM1_ESM.pdf]

Supplementary information

Cerebral ischemia induces TRPC6 via HIF1α/ZEB2 axis in the glomerular podocytes and contributes to proteinuria

Krishnamurthy Nakuluri[¶*], Rajkishor Nishad[¶*], Dhanunjay Mukhi[¶], Sireesh Kumar[§], Venkata P Nakka[$,] Lakshmi P Kolligundla[¶], Parimala Narne[§], Sai Sampath K Natuva[♯], Prakash Babu Phanithi[§], Anil K Pasupulati[¶]

[¶]Department of Biochemistry, University of Hyderabad, Hyderabad, India-500046
[§]Department of Biotechnology & Bioinformatics, University of Hyderabad, Hyderabad, India-500046
[$]Department of Biochemistry, Acharya Nagarjuna University, Guntur, India-522510
[#]Narayana Medical College, Nellore, India-524003

Figure S1: Elevatees treated with FG-4592. Differentiated human podocytes were treated with FG-4592 for 24 hrs and immunostaining was performed for TRPC6 and images were captured with LeicaTrinocular microscope (Scaling 50 μm).

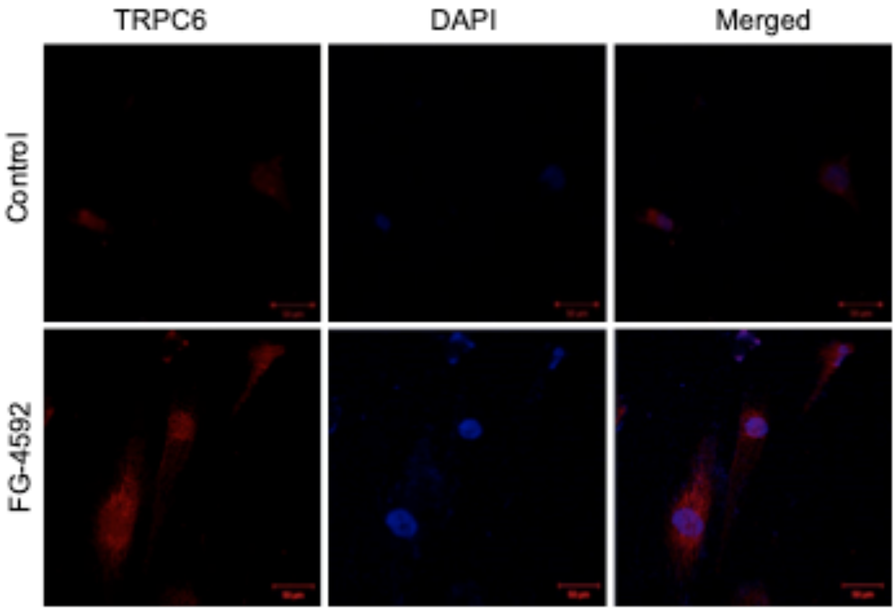

Figure S2: Elevated levels of pFAk in podocytes treated with FG-4592. Differentiated human podocytes were treated with FG-4592 for 24 hrs and immunostaining was performed for pFAK and images were captured with Leica Trinocular microscope (Scaling 50 μm).

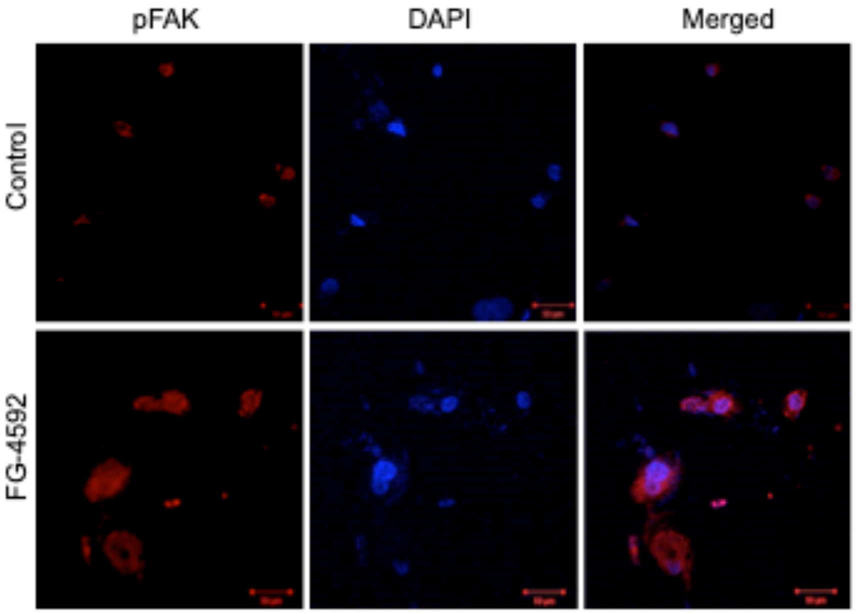

TableS1: List of primers used for RT-PCR

Human	Forward Primer	Reverse Primer
ZEB2	AGTGTGCCCAACCATGAGTC	GGTCTGGATCGTGGCTTCTG
TRPC6	AAGGGTGCTAGGATTGAACG	CTAGACAATGACAGGTAAGCCG
HIF1α	ATCATATCACTGGACTTCGGC	AGTTTCAGAGGCAGGTAATGG
Rat		
ZEB2	GAGATAAGGGAGAGCGTTGTG	AATTGTGGTCTGGATCGTGG
TRPC6	GGTTATGTACGGATTGTGGAGG	ACATCGTGGGAGAATCTTGTC
HIF1α	ATCATATCACTGGACTTCGGC	AGTTTCAGAGGCAGGTAATGG

TableS2: List of primers used for ChIP assay

	Forward Primer	Reverse Primer
ZEB2	TCATTAGAAGAGGCGTAACAC G	GACCGTTATTCCTGCAGAGC
E-Cad	GAGGGTCACCGCGTCTATGC	GGGCTGGAGTCTGAACTGAC
VEGF	GCGTGTCTCTGGACAGAGTTT	AGCCTCAGCCCTTCCACA
TRPC6 control	CCACCTGTTCATGGGAAGC	TTGATGTGATGGCAGCTGGT
TRPC6 FP1	GAAACGCAGTTGGCATTCATT	TTCCTTCCGTCACTGCGAA
TRPC6 FP2	ACGAAGTCCGCAGTTGGTA	GAGACTCTCCAGCCCTCAGCTC
TRPC6 Promoter	TCGACGCGTCAGATAGCAGTT GCCACAAAA	GGAAGATCTAGCGAAGCGTAAGAG CGGAGA
